# Why Is Virtual Reality Interesting for Philosophers?

**DOI:** 10.3389/frobt.2018.00101

**Published:** 2018-09-13

**Authors:** Thomas K. Metzinger

**Affiliations:** ^1^Philosophisches Seminar, Johannes Gutenberg-Universität, Mainz, Germany; ^2^Frankfurt Institute for Advanced Studies, Frankfurt am Main, Germany

**Keywords:** virtual reality, augmented reality, mixed reality, emptiness, philosophy of religion, life-world, social hallucinations, consciousness

## Abstract

This article explores promising points of contact between philosophy and the expanding field of virtual reality research. Aiming at an interdisciplinary audience, it proposes a series of new research targets by presenting a range of concrete examples characterized by high theoretical relevance and heuristic fecundity. Among these examples are conscious experience itself, “Bayesian” and social VR, amnestic re-embodiment, merging human-controlled avatars and virtual agents, virtual ego-dissolution, controlling the reality/virtuality continuum, the confluence of VR and artificial intelligence (AI) as well as of VR and functional magnetic resonance imaging (fMRI), VR-based social hallucinations and the emergence of a virtual *Lebenswelt*, religious faith and practical phenomenology. Hopefully, these examples can serve as first proposals for intensified future interaction and mark out some potential new directions for research.

“Virtual reality encompasses virtual *un*reality” (Slater and Sanchez-Vives, 2016, p.38).

## Introduction

What are the most promising future directions for an intensified cooperation between the philosophical community and virtual reality research (VR), potentially also including other disciplines like cognitive neuroscience or experimental psychology? The purpose of this contribution is to take a fresh look, from a philosopher's perspective, at some specific research areas in the field of VR, isolating and highlighting aspects of particular interest from a conceptual and metatheoretical perspective. This article is intended as a source of inspiration for an interdisciplinary audience; if each reader finds just one of the ideas presented below useful, it will have served its purpose. Hence the article was not written as a technical contribution by one philosopher for other philosophers and is not meant as an exhaustive list of philosophical research targets. I simply draw attention to a selection of topics that are, I believe, characterized by an exceptionally high degree of heuristic fecundity. To make these issues accessible to an interdisciplinary readership, I will briefly introduce some central concepts as I go (see Box 1), and sometimes use a more essayistic style. The hope is that these topics, deliberately presented along with a series of concrete examples, can serve as contact points between both disciplines and mark out promising subfields in which VR researchers and the philosophical community could profit from intensified future interaction. I will briefly highlight the theoretical relevance of most examples, along with the potential future benefits of intensified cooperation. Sometimes, I will also try to sketch a specific technological realization that would interestingly constrain philosophical theory formation, open new routes, or constitute the “perfect” or “maximal” VR-experience in a given context.

Box 1 **Philosophical concepts.**

**Amnestic re-embodiment**

Re-embodiment of the subject of experience in VR without the conscious knowledge *that* one is currently immersed in a virtual environment and identified with a virtual body or character.

**Counterfactual content**

A linguistic statement or a mental representation has counterfactual content if it contradicts the current state of reality. Thought experiments, conscious experiences, and most computer-generated models of reality are counterfactual in this sense, because they do not represent or reflect the actual, current state of the world. In some cases, they may simply be classified as “false” or “misrepresentational,” in other cases they may be adequate, for example if they target a *possible*, but highly likely perceptual situation.

**Epistemic agent model (EAM)**

A conscious internal model of the self as actively selecting targets of knowledge, as an agent that stands in epistemic relations (like “perceiving,” “believing,” “knowing”) to the world and to itself (as in “controlling the focus of attention,” “reasoning” or “knowing that one knows”), and as an entity that has the *capacity* to actively create such relations of knowing. Human beings only have an EAM intermittently, for about one third of their conscious life-time (Metzinger, 2013b, Metzinger, 2015 section 2.5, in Metzinger, 2017). Today's virtual agents and robots are not yet driven by an internal EAM.

**Epistemic innocence**

The theory that certain mental processes such as delusion and confabulation (which may count as suboptimal from an epistemological perspective) can have epistemic *benefits*. The idea is that in some cases, what superficially appears as an imperfect cognitive process may really enable knowledge acquisition.

**Epistemology**

The study of knowledge that seeks to answer questions like: What are the necessary and sufficient conditions for saying that one possess knowledge? What makes a belief a *justified* belief? How many different kinds of knowledge are there? Is there anything like certainty?

**Global neural correlate of consciousness (GNCC)**

The minimally sufficient set of neurofunctional properties that brings about the conscious model of reality as a whole at a given point in time.

**Global transparency**

Phenomenal transparency for a whole conscious model of reality.

**Hybrid avatar/virtual agent systems (HAVAS)**

Digital representations of persons and/or epistemic agents which are simultaneously human-controlled and AI-controlled.

**Justified true belief**

According to one traditional philosophical model, three individually necessary conditions (namely, truth, belief, and justification) are jointly sufficient for a subject *S* to possess knowledge: *S* knows that *p* if and only if *p* is true and *S* is justified in believing that *p*.

***Lebenswelt*** **(life-world)**

A pre-given social world in which subjects experience themselves as being united by a quality of “togetherness.” A *Lebenswelt* is intersubjectively given and is actively constituted by everyday social interactions leading to a shared first-person plural perspective (a more or less implicit group context, a mentally represented “we”).

**Ontology**

In philosophy, the investigation of *what there is*, i.e., of what entities exist and what the most general features and relations of those entities are (for example, physical objects, God, universals, numbers, etc.). In computer and information science, the representation, formal naming, and definition of entities and relations substantiating a domain.

**Other-minds illusion**

The conscious experience of currently interacting with a system that has mental states when it really has none, for example the (hallucinatory) experience of encountering another self-conscious entity that actively selects targets of knowledge or is really “perceiving,” “believing,” “knowing” in a way that is relevantly similar to the observer. An other-minds illusion is a social hallucination.

**Other-minds problem**

The epistemological problem of gaining knowledge about another entity's mental states, for example the subjectively felt character of its conscious experiences or the content of its beliefs.

**Phenomenal transparency**

Transparency as used in this article is a property of conscious representations; unconscious representations in the human brain are neither transparent nor opaque. “Transparency” means that only the content of a representation is available for introspective access; the earlier processing stages or aspects of the construction process are hidden. Therefore, the content cannot be subjectively experienced *as* a representation. This leads to the phenomenology of “direct realism,” a subjective experience of immediacy and realness, as if, for example, directly perceiving mind-independent objects.

**Phenomenal unit of identification (UI)**

The conscious content that is referred to in phenomenological reports of the type “I *am* this!” (see section Example 2: Embodiment and Bodily Self-Consciousness, for a definition cf. Metzinger, 2018a).

**Postbiotic social boot-strapping scenario (PSBS)**

A scenario in which multiple AIs create a non-biological *Lebenswelt* by mutually interacting with each other using virtual agents based on transparent, VR-based personoid interfaces, thereby causing robust other-minds illusions in each other. Such systems would apply the algorithms they originally developed in man-machine interactions to machine-machine communication, while still using individual virtual avatars or agent-models as their interfaces.

**rt-fMRI-NCCF**

A real-time fMRI representation of the global NCC that is directly converted into a virtual reality environment. This would create a perceivable dynamic landscape which the conscious subject can directly experience, navigate, and causally influence via multiple real-time neurofeedback loops.

**Second-order virtual agent**

A machine-controlled virtual character or person-model that transparently represents itself as socially situated, i.e., that has an internal model of itself as standing in genuine social relations to other persons or other self-conscious agents. A second-order agent has an inbuilt other-minds illusion.

**Social hallucination**

See other-minds illusion.

**Synthetic phenomenology (SP)**

Artificial conscious experience realized on non-biological carrier systems.

## Philosophy of mind

Empirically informed philosophy of mind is, rather obviously, the area within philosophy that can most directly profit from recent results in VR research. The VR community should also actively seek more productive input from philosophers of mind. I will confine myself to two examples.

### Example 1: consciousness

The richest, maximally robust, and close-to-perfect VR-experience we currently know is our very own, ordinary, biologically evolved form of waking consciousness *itself*. VR is the best technological metaphor for conscious experience we currently have. The history of philosophy has shown how technological metaphors for the human mind always have their limitations: Think of the mechanical clock, the camera, the steam engine, or, more recently, the computer as a physically realized abstract automaton, with psychological properties as exhaustively described by a Turing machine table (Putnam, 1967, 1975, 1992; Churchland, 2005; Boden, 2006). All these metaphors have severe limitations. Using the computer example, the classical-cognitivist metaphor of a von-Neuman-machine cannot accommodate dynamical embodiment, subsymbolic representation, non-rule based types of information processing, or the experiential character of phenomenal states as subjectively experienced from a first-person perspective. Nevertheless, it is hard to underestimate the influence and impact the long-abandoned “computer model of mind” has had on modern analytic philosophy of mind. Technological metaphors often possess great heuristic fecundity and help us in developing new ideas and testable hypotheses. Indeed, the computer model of mind has led to the emergence of a whole new academic discipline: classical cognitive science. Similarly, I believe that the heuristic potential of the VR metaphor for philosophical theories of consciousness has just barely been grasped.

Some philosophers (Metzinger, 1991, p. 127; Metzinger, 1993, p. 243; Metzinger, 2003a, 2010, p. 6; Revonsuo, 1995, p. 55; Revonsuo, 2006, p. 115; Noë, 2002; cf. Clowes and Chrisley, 2012; Westerhoff, 2016, for critical discussion and recent overviews) have already argued at length that the conscious experience produced by biological nervous systems *is* a virtual model of the world—a dynamic internal simulation. In standard situations it cannot be experienced *as* a virtual model because it is phenomenally transparent—we “look through it” as if we were in direct and immediate contact with reality (Moore, 1903; Metzinger, 2003b; for the notion of “phenomenal transparency” and a brief explanation of other philosophical concepts see Box 1). Likewise, technological VR is the representation of *possible* worlds and *possible* selves, with the aim of making them appear ever more realistic—ideally, by creating a subjective sense of “presence” in the user. “Presence” is a complex phenomenal quality, the three major dimensions of which are *identification* (i.e., being present *as a self*), *self-location in a temporal frame of reference* (i.e., being present as a self *now*, in this very moment), and *self-location in space* (i.e., the classical “place illusion,” Slater and Sanchez-Vives, 2016). “Presence” is a phenomenal quality normally going along with a minimal sense of selfhood (Blanke and Metzinger, 2009), and it results from the simulation of a self-centered world—in VR settings as well as in everyday life. Interestingly, some of our best theories of the human mind and conscious experience itself use a similar explanation: Leading current theories of brain dynamics (Friston, 2010; Hohwy, 2013; Clark, 2016; Metzinger and Wiese, 2017) describe it as the constant creation of hierarchical internal *models* of the world, virtual neural representations of reality which express probability density functions and work by continuously generating hypotheses about the hidden causes of sensory input, minimizing their prediction error (see Wiese and Metzinger, 2017 for an accessible introduction). The parallels between virtuality and phenomenality are striking. Here are some points of contact between VR and philosophical phenomenology:

- Phenomenal content and virtual content are both counterfactual.
  Our best current theories of consciousness describe it as something that could be called a form of “online dreaming” (Metzinger, 2003a, p. 140), in which conscious waking is a dreamlike state currently modulated by the constraints produced by ongoing sensory input. It is a controlled hallucination based on *predictions* about the current sensory input (Hohwy, 2013; Clark, 2016; Wiese and Metzinger, 2017). Relative to the actual state of the world, if taken as *referring* to this state, all predictive representations are non-veridical. Strictly speaking they are misrepresentations—but are nevertheless potentially beneficial for the system in which they occur (Wiese, 2017). VR content is typically part of an animated computer graphics model, and if taken as depicting the actual physical 3D scene surrounding the user, it is also a misrepresentation. However, VR content does not result from a design flaw—the whole point is to generate perceptual representations of *possible* worlds in the user's brain, not of the actual one. Phenomenal content (the brain-based content of conscious, subjective experience) is the content of an ongoing simulation too: a prediction of the *probable* causes of a sensory signal. It is not a veridical representation of the actual environment, and it is useful for precisely this reason. By definition, machine-generated virtual content is counterfactual (see Box 1) as well, although it may be interestingly blended with real-world elements, as in augmented reality (AR) setups.

If this first point is correct, then it would be interesting to create VR utilizing the same mechanisms the human brain uses. What if, for example, in dynamically updating itself, the animated computer graphics model used the same computational principles of top-down processing, statistical estimation, prediction error minimization, hierarchical Bayesian inference, and predictive control many theoreticians now believe to be operative in the brain itself? Would this change the user's phenomenology in any interesting way, for example its fine-grained temporal dynamics? This is one example of a new research question that is interesting from a philosophical perspective, but which also has implications for making the VR experience better. It should therefore be of interest to people in the field and it could be tackled with interdisciplinary cooperation.

- VR and conscious experience both present us with an integrated ontology.
  Ontology is not only a subfield in academic philosophy investigating the logic and semantics of concepts like “being,” “becoming,” or “existence.” The concept also refers to an area in computer science and information science investigating the representation, formal naming, and definition of the categories, properties, and relations of the concepts, data, and entities that substantiate a given—or even all possible—domains. Interestingly, the conscious brain is an information-processing system too, and it certainly represents data and entities as “being,” “becoming,” or “existing.” Conscious experience can be described as a highly-integrated set of hypotheses about the likely causes of the inputs received by the embodied brain, in the external as well as in the internal (i.e., intraorganismic) environment. It is not a list of propositions containing existential quantifiers. For an information-processing system to be conscious means it runs under an integrated ontology (see Box 1), a unified, subsymbolic situation model, which is internally presented to it in an integrated temporal frame of reference defining a subjective now, a “window of presence” (Metzinger, 2003a). VR creates ontologies and integrated situation models too, but their presentation within a single “lived moment” (i.e., a Jamesian “specious present,” the temporal frame of reference referred to above, plus the construction of an experiential subject; see Clowes and Chrisley, 2012, p. 511), is still left to the brain of the user. If this is correct, then it follows that if we understand the computational principles underlying self-location and self-presentation within an internal temporal frame of reference in our brains, and if future VR technology were then to create a virtual “specious present” as part of a yet-to-be-invented form of virtual time representation, then this would amount to the creation of a very simple form of artificial consciousness.

The conjunction of these two first points leads us to a bold general claim: The “perfect” VR system would lead to artificial consciousness—the creation of synthetic phenomenology (SP; see Box 1)^1^. Call this the “SP-principle”: In its maximal realization, VR would be tantamount to the creation of artificial phenomenal states, to a technological realization of synthetic phenomenology. Of course, this would have to include a fully integrated multimodal scene, a virtual “specious present,” a self-model that creates a first-person perspective by being a virtual model of an epistemic agent (EAM; see Box 1 and section 2.5 in Metzinger, 2017, for details and further references), plus global transparency (see Box 1). Today, there still is a biological user, partially immersed in a visual situation model created by advanced computer graphics, and what VR technology ultimately aims at is real-time control of the information flow within the minimally sufficient global neural correlate of consciousness (GNCC; see Box 1).

This seems to be a second general principle: As of today, the ultimate “engineering target” is the conscious model of reality in a biological agent's brain. VR is a non-invasive form of neurotechnology targeting the GNCC. But imagine a world without conscious biological creatures, in which an autonomous, intelligent robot had learned to control its interaction with its physical environment by opening an internal global workspace. Then imagine that the content of this workspace is determined by the “perfect” VR sketched above. If we imagine this robot as *internally* using a maximal realization of VR—global integration, specious present, transparency, and a self-model which it now “confuses” with itself (see section Example 2: Embodiment and Bodily Self-Consciousness and Metzinger, 2003a), then this would be tantamount to a machine model of embodied conscious experience.

- Phenomenal content and virtual content are both locally determined.

It is widely accepted in philosophy of mind that phenomenal properties supervene locally; and as of today, virtual content is processed in single machines and presented by local devices to individual users. This will soon change through the confluence of developments in VR, brain-computer interfaces (BCIs), and social networks. For philosophers, this will create an interesting new target for the internalism/externalism debate on mental content (Menary, 2010). For computer scientists, the question arises of what the “perfect” form of social VR would actually be. In social VR, what exactly is the relationship between the phenomenal content locally instantiated in the brains of multiple users and shared virtual content created by causal interactions distributed over different machines and artificial media? Social VR is a field that needs a combination of new technological approaches and rigorous conceptual analysis, for instance with regard to the concept of “tele-immersion” (for an excellent example, see Ohl, 2017).

**Figure 1:**
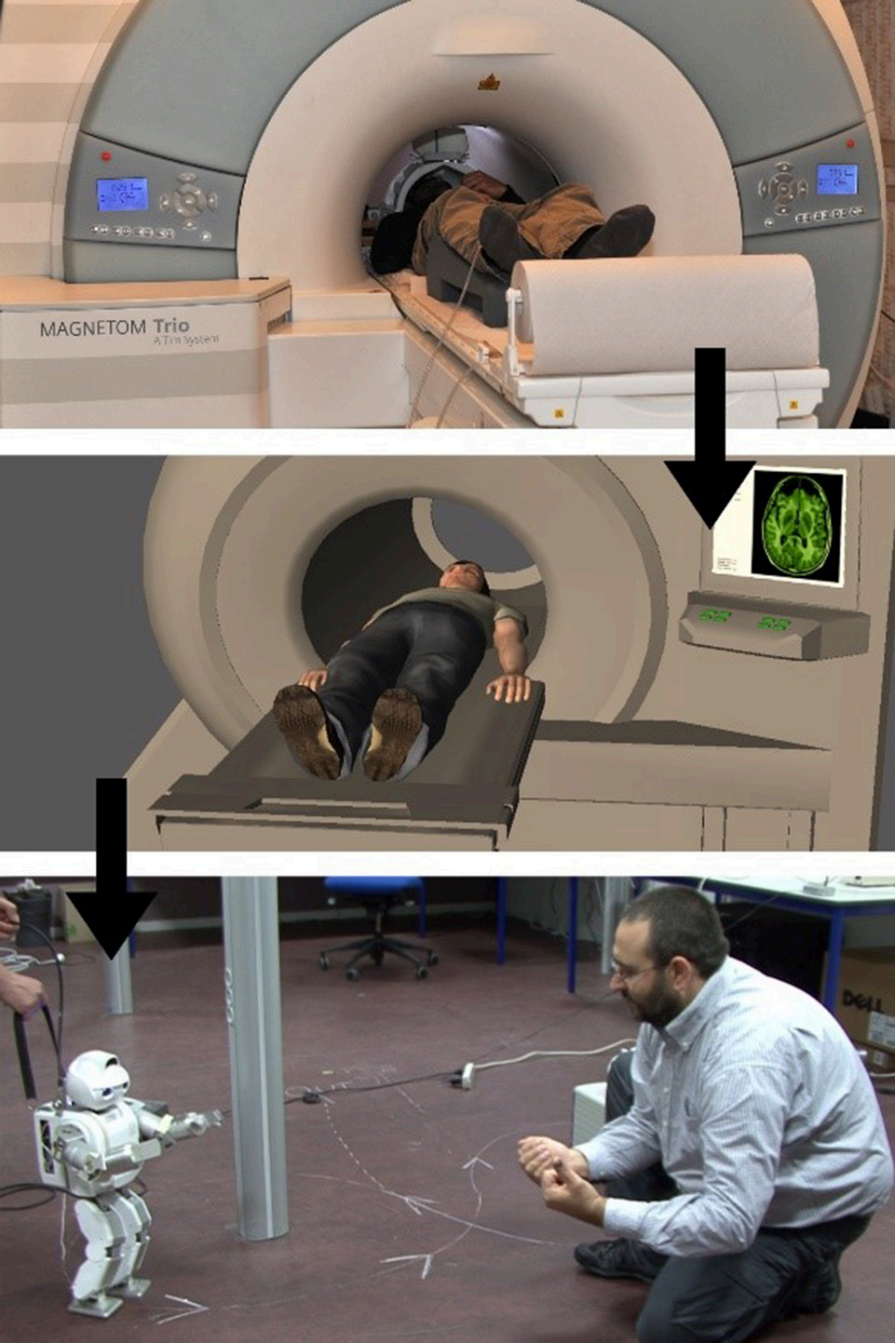
“PSM-actions”: A test subject lies in a nuclear magnetic resonance tomograph at the Weizmann Institute in Israel. With the aid of data goggles, he sees an avatar, also lying in a scanner. The goal is to create the illusion that he is embodied in this avatar. The test subject's motor imagery is classified and translated into movement commands, setting the avatar in motion. After a training phase, test subjects were able to control a far remote robot in France “directly with their minds” via the Internet, seeing the environment in France through the robot's camera eyes. Figure with friendly permission from Doron Friedmann and Michel Facerias; written informed consent for publication of figure has been provided by Michael Facerias.

The first contribution computer scientists can and certainly will make lies in the field of *interface design*: For the special domain of social VR, what would be the most efficient and reliable interfaces linking human brains via BCI-coupling and shared VR? This issue is theoretically relevant because it addresses a classical philosophical problem: the “other-minds problem” (Box 1). We assume that each of us has a direct knowledge of our own experience, but we can never directly know that someone other than ourselves is in the mental state they are in. Could social VR create more direct forms of knowing another person's mind? Could it provide us with new means of acquiring phenomenological concepts like “red” or “joy” which we apply to inner states of sentient creatures other than ourselves? We have already begun to causally couple the self-models in human user's brains to robots and avatars via robotic and virtual re-embodiment today (see Figure 1), but what would a more direct linkage of conscious minds involve? They could constitute new inner “modes of presentation” for social facts, as philosophers might say. It is interesting to note how we are already beginning to re-embody ourselves not only in robots and avatars, but also in other human being's physical bodies (De Oliveira et al., 2016). This naturally leads to the question of virtually re-instantiating the higher levels of a human user's self-model in those of another human being's self-model, of a more abstract form of re-embodiment in another self-conscious *mind*. This then would be the step from virtual body swap (Petkova and Ehrsson, 2008) to virtual mind swap. Apart from a careful conceptual description of research targets, the coupling of whole conscious world-models plus embedded high-level, cognitive self-representations (and not only *bodily* self-models) would require a deep confluence of neurotechnology and VR. The maximal realization of social VR would therefore consist in creating an artificial platform on which whole individual biological minds can *merge*, thereby transcending the principle of local determination.

- Phenomenal content and virtual content can vary along a continuum of opacity and transparency, respectively, of *explicit* virtuality and projected realism.
  Today, a broad standard definition of “phenomenal transparency” (Box 1), on which most philosophers roughly agree, is that it essentially consists in only the *content properties* of a conscious mental representation being available for introspection. Any non-intentional or “vehicle-properties” involved in the representation are not available for introspection. In other words, it is not experienced *as a representation*. Introspectively, we can access its content, but not the content-formation process itself. Typically, it is assumed that transparency in this sense is a property of *all* phenomenal states (for more, see Metzinger, 2003a,b).

But of course, the standard assumption is incomplete, because *opaque* phenomenal representations also exist (whereas unconscious states are neither transparent nor opaque in this sense). Phenomenological examples of opaque state-classes are, most notably, consciously experienced thoughts: We experience them as mind-dependent, as mental representations that could be true or false. Similarly, some emotions, pseudo-hallucinations, and lucid dreams are subjectively experienced *as representational processes*. Most importantly, the phenomenology of VR is also typically characterized by incomplete immersion, with varying degrees of opacity. This may change as the technology advances. Phenomenally opaque processes sometimes appear to us as deliberately initiated cognitive or representational processes. However, sometimes they appear to be automatic or spontaneously occurring; they are limited or even global phenomenal simulations and frequently are not under the experiential subject's control.

Here is another concrete proposal: Perhaps the most interesting contribution VR researchers could make is to develop a reliable “volume control for realness.” Obviously, a clear conceptual taxonomy is needed as well, but the role of computer scientists in this type of cooperation would lie in developing a metric for immersion and self-identification—a quantifiable approach. The interesting point here is that human phenomenology varies along a spectrum from “realness” to “mind-dependence.” This frequently overlooked phenomenological feature provides another conceptual bridge into the representational deep structure of VR-environments: there are *degrees of immersion*. VR environments can be more or less realistic, and this general property is itself directly and concretely reflected in the user's phenomenology (cf. the epigraph for this article). Below I will argue that VR is the most relevant technology to create innovative experimental designs for philosophical phenomenologists interested in empirically researching the transparency/opacity continuum characterizing human consciousness (as introduced in Metzinger, 2003b).

The “perfect” form of VR technology would be one in which the user—or the experimental psychologist, neuroscientist, or philosopher interested in consciousness—could reliably set the “level of realness” for the experience. If we calibrate the transparency parameter of ordinary waking states as 1, then possible levels would include values >1, leading to “hyperreal” phenomenologies (as in certain drug-induced states of consciousness, during “ecstatic” epileptic seizures, or religious experiences), and values < 1 (as in “unreal” experiences like depersonalization or derealization disorder). Some VR applications aim at phenomenal presence, realism, embodiment, and an illusion of immediacy, others will want to create a “dreamlike” quality (for example in entertainment settings). There are two specific subtypes of phenomenal states which (a) are of special systematic interest to philosophers, and (b) are directly related to the typical phenomenological profile created by VR technology: the lucid dream state and the out-of-body experience (OBE; see Metzinger, 2009b, 2013c, for philosophical discussion).

There has been a lot of excellent work trying to create OBEs in VR labs, trying to make it a repeatable, experimentally controllable phenomenon (see Ehrsson, 2007; Lenggenhager et al., 2007 for classical studies; Blanke, 2012, for a review). So far, these attempts have not been successful because users do not yet look out of the eyes of the avatar offered as an alternative unit of identification (UI, which in this and other articles is *not* an abbreviation for “user interface,” but instead refers to the conscious experience of self-identification; see Box 1, Metzinger, 2018a and the next section for a definition of the concept). Rather, the resulting phenomenology typically resembles the clinical phenomenon of heautoscopy. According to the self-model theory of subjectivity (SMT; Metzinger, 2003a, 2008), the main reason for this failure is that the user's “interoceptive self-model” is firmly locked in the biological body; it cannot be simulated in an avatar yet. The interoceptive self-model is that layer of bodily self-representation in the brain that is driven by internal signals from the viscera and other areas signaling the state of the body to the brain (Craig, 2009; Barrett and Simmons, 2015). The prediction under SMT is that full identification with an avatar can only be achieved under two conditions: Either the avatar has its own interoceptive self-model that can be synchronized with the biological counterpart in the user's brain, or interoceptive experience is selectively blocked and *another* artificial unit of identification (Box 1) is created and technologically exploited. According to SMT, a prime candidate would be the sense of effort going along with mental forms of agency like controlling one's own attention, because this is what creates the sense of self on the mental level. The empirical prediction from SMT is that if avatars in virtual reality had a functional analog of visual attention which the user could control, then the consciously felt “sense of effort” of the user trying to control the avatar's attention would create a deep form of identification. This is another concrete research proposal, derived from a philosophical theory, but fully testable and open for interdisciplinary cooperation. We could call the proposed strategy “subjective identification via interoceptive extinction plus synchronized attentional agency.”

What about creating not OBEs (which involves an externalized visuospatial perspective), but lucid dreams with the help of VR technology? The dream body can also be completely devoid of an interoceptive self-model. In section Example 1: Consciousness, I said that waking consciousness could be called a form of “online dreaming.” Could VR help to create a new, distinct class of phenomenal states in the form of a new version of *lucid* online dreaming? Having a metric and an implemented, quantifiable “realness control” for VR would enable experimental psychologists to create a machine-model of the lucid dream state. It would be highly interesting for philosophers of mind, dream researchers, and phenomenologists if they could use VR technology to explore the transparency/opacity gradient of their very own conscious experience at will.

In both subtypes, certain content elements may be experienced as only virtual (e.g., dream reality as such or the immediate environment in which an OBE unfolds), while, phenomenologically, others remain as ultimately real (for example, even in lucid dreams other dream characters encountered by the experiential subject are often taken to be real entities, as is the transparent model of the knowing, observing self in an OBE). On the technological side, the reality/virtuality continuum encompasses all possible variations and combinations of real and virtual objects (Milgram et al., 1994; Milgram and Colquhoun, 1999). The “reality/virtuality continuum” has been described as a concept in new media and computer science, but it is interesting to note how our very own everyday phenomenology also possesses elements that appear “unreal” to us (optical illusions, benign pseudo-hallucinations) or as only diffusely or not at all located in physical space, e.g., as “unworldly,” “disembodied,” or “mental” (namely, mental action and mind-wandering; see Metzinger, 2015, 2017).

However, as philosophers we must never forget that the reality/virtuality continuum itself only appears in a virtual model activated by our brain. In addition, this brain is embodied and developed against the historical-cultural context of the cognitive niche in which we are born. This is, unfortunately, a deep structural feature which systematically hides its own virtuality from its user, the biological organism in which it appears. One philosophically interesting point is that investigating the phenomenology of VR will give us a deeper understanding of what it really means—and why it was functionally adequate—that the reality-appearance distinction became attentionally as well as cognitively available by being represented *on the level of appearance* (Metzinger, 2003a). It also leads to subtle and potentially novel insights into the specific phenomenal character related to metaphysical indeterminacy (see section VR-Phenomenology in the Context of Comparative and Transcultural Philosophy).

### Example 2: embodiment and bodily self-consciousness

Advanced VR technology seeks not only to create the classical place illusion described by Slater and Sanchez-Vives (2016) (section Introduction), it also increasingly targets the deepest layers of human self-consciousness by utilizing techniques for virtual embodiment and robotic re-embodiment (Cohen et al., 2012, 2014a,b; see vereproject.eu for further examples). We are already beginning to use VR technology for re-embodiment in other human bodies (De Oliveira et al., 2016) and many of the more recent empirical results are highly interesting from a conceptual and metatheoretical perspective (see Ehrsson, 2007; Lenggenhager et al., 2007, for classical studies; Metzinger, 2008, 2009a,b, for accessible introductions; Blanke, 2012, for a review). First, they allow us to distinguish different levels of embodiment and to develop a more fine-grained analysis of bodily self-awareness in humans; second, they open the door to a deeper understanding of the *mechanism of identification* underlying the way in which a conscious subject of experience locates itself in time and space by identifying with a body. Let me briefly explain this point, as it is of interest for philosophers.

Let us say that for every self-conscious system *S* there exists a **phenomenal unit of identification** (UI, Box 1) such that

- *S* possesses a single, conscious model of reality;
- the UI is a part of this model;
- at any given point in time *t*, the UI can be characterized by a specific and determinate representational content *C*;
- such that *C* constitutes the transparent part of the system's phenomenal self-model (PSM; Metzinger, 2003a) at *t*.

If we assume a “predictive processing” model of human brain activity (Friston, 2010; Hohwy, 2013; Clark, 2016; Metzinger and Wiese, 2017), then, for all human beings, *C* is always counterfactual content because it does not refer to the currently present, actual state of the world. The UI is the best hypothesis the system has about its own global state (Limanowski and Blankenburg, 2013; Limanowski, 2014). For human beings, *C* is dynamic and highly variable, and it need not coincide with the physical body as represented (for an example, see de Ridder, 2007). There exists a minimal UI, which is likely constituted by pure spatiotemporal self-location (Blanke and Metzinger, 2009; Windt, 2010, 2017; Metzinger, 2013b,c); and there is also a maximal UI, likely constituted by the most general phenomenal property available to *S* at any point *t*, namely, the integrated nature of phenomenality *per se* (Metzinger, 2013b,c, 2016). *C* is phenomenally transparent: Internally, *S* experiences the representational content constituting the UI neither as counterfactual nor as veridical, but simply as real. Phenomenally experienced realness is an expression of successful prediction error minimization, high model evidence, and counterfactual richness (e.g., invariance under counterfactual manipulation). Therefore, the UI simply is the transparent partition of the PSM^2^. I submit that perhaps the central philosophical relevance of recent work on virtual embodiment and robotic re-embodiment is that it holds the promise of introducing a set of more fine-grained conceptual distinctions into the theory of embodiment and self-consciousness. Could VR researchers also create a “volume control for self-identification”? Work in VR that helps us to experimentally manipulate the UI in a non-invasive but causally fine-grained manner has already successfully demonstrated its relevance for the neuroscience of bodily self-consciousness, for example by creating innovative experimental designs. Philosophers have already cooperated with neuroscientists and shown how the UI can be influenced to “drift toward” an avatar and how peripersonal space can be expanded in a VR-setting (Blanke et al., 2015; Noel et al., 2015; Serino et al., 2015). However, there are two logical steps that have not yet been taken. There are two types of experiment that would be of great interest to philosophers of mind: *Maximizing* the UI, and *deleting* the UI from human phenomenal space altogether. The open question is if engineers and scientists in VR could technologically implement this.

How would one create a VR experience in which the user becomes one with everything? Clearly, this would have to be an entirely passive experimental setup, because any bodily or mental interaction of the user with the system would immediately create a felt sense of agency and therefore keep its phenomenal model of reality split into subject and object, divided into a knowing self and an external environment. How could one create an entirely passive VR experience in which *everything* the user experiences gradually turns into one big knowing self, a single conscious unit of identification that has been maximized by being expanded to the boundaries of the phenomenal world?

Experiments of the second type would aim at creating selfless states of consciousness. Instead of ego-expansion they would aim at ego-elimination. Such experiments would be interesting because they would create a contrast class or a set of alternative experiences not characterized by a UI—states without any consciously experienced ego. The phenomenon of “ego dissolution” is well-known from pharmacological interventions by classical psychedelics, dissociative anesthetics and agonists of the kappa opioid receptor (Millière, 2017) and it can be measured, for example by the Ego-Dissolution Inventory (EDI; Nour et al., 2016). It occurs in psychiatric diseases, and it has also been reported across the centuries by spiritual practitioners from many different cultures. Comparing results from both types of experiments might help to decide the question if the existence of a UI *necessarily* leads to a consciously experienced sense of self, or if some states created by maximizing the UI are actually selfless states if assessed with the help of existing inventories for measuring the degree of ego-dissolution. Here, one central question—highly relevant for philosophers, psychologists, and neuroscientists alike—is whether research in VR could help to establish a double dissociation between the phenomenology of identification and the phenomenology of selfhood.

## Epistemology

Epistemology is the study of knowledge and is concerned with questions such as: What are the necessary and sufficient conditions for the possession of knowledge? How many kinds of knowledge are there, and what is their structure, what are their sources and boundaries? What makes a belief a *justified* belief (Box 1)? Accordingly, VR-epistemology might ask questions like these: How does one obtain knowledge about virtual objects, and how do we arrive at justified beliefs about facts holding in a virtual world? Are there such things as *virtual facts*? Are the necessary and sufficient conditions of knowledge interestingly different if we limit our domain to perceptual content presented via VR? What are the sources of knowledge about elements of a given virtual world? Is justification relative to this specific class of epistemic objects internal or external to one's own mind?

### Example 3: amnestic re-embodiment and epistemic innocence

VR settings immediately remind every philosopher of Descartes' dream argument: Even in a best-case scenario of sensory perception, we can never rule out that we are now dreaming, because dreaming is subjectively indistinguishable from waking experience (see Windt, 2015, section Example 1: Consciousness). If classical Cartesian Dream Skepticism is on the right track, then at any given moment our implicit belief that we are awake might be mistaken. If this basic background assumption is correct, our current belief that we are, at this moment, *not* in VR might be mistaken as well, although “in order for the VR to be indistinguishable from reality, the participant would have to not remember that they had “gone into” a VR system” (Slater and Sanchez-Vives, 2016, p. 37).

One interesting form of collaboration between philosophers and VR researchers would be to systematically transpose classical philosophical thought experiments into VR-settings. Here, the question would be if VR researchers could create a full-blown “Cartesian dream.” Could there be something like “amnestic re-embodiment” (see Box 1) in VR? It seems there are many conceivable scenarios of VR use in which this constraint (let us call it the “SSV-constraint,” as it was introduced by Slater and Sanchez-Vives) could be satisfied, for example in animals equipped with head-mounted displays, in human children, in drug users, in sleep labs, or in patients suffering from severe amnesia, intoxication syndromes, or dementia. Moreover, in future entertainment scenarios or in therapeutic applications it may exactly become a goal to purposefully satisfy the SSV-constraint, to make users *forget* the fact that they currently are in VR.

Bortolotti (2015a,b) recently introduced the concept of “epistemic innocence” (Box 1) to articulate the idea that certain mental processes such as delusion and confabulation (which may count as suboptimal from an epistemological perspective) may have not just psychological, but also epistemic benefits. Perhaps amnestic re-embodiment in VR (say, in a pharmacologically supported therapeutic context) could lead not only to psychological benefits that are not simply purchased with the epistemic cost of episodic amnesia, but which also causally enable forms of genuine *knowledge acquisition*, for example new forms of self-knowledge. Perhaps new philosophical ideas like “amnestic re-embodiment” or “epistemic innocence” can be fruitfully applied in the domain of VR if actually implemented and viewed as a tool for self-exploration, cognitive enhancement, or future psychotherapy. And of course, on a speculative metaphysical level, it is only a question of time until the SSV-constraint will be discussed in relation to real-life experience, to traditional religious theories of reincarnation, “pre-birth amnesia,” etc. In any case, it seems safe to predict that many classical issues of external-world skepticism may re-appear in a new guise, playing a central role for the new discipline of VR epistemology.

### Example 4: knowing personal identity

Here, I name one particular example of great relevance for the philosophy of law and the applied ethics of VR: The problem of reliably knowing about another human agent's *personal identity* in VR. If I want to reliably interact with another human being in VR, for example via avatar-to-avatar interaction, then I need to know the identity of the person currently controlling or even phenomenologically identifying with that avatar. This leads to the problem of avatar ownership and individuation, which will certainly be an important future issue for regulatory agencies to consider.

*How does one assign an unequivocal identity to the virtual representation of a body or a person? Could there be something like a chassis plate number, a license plate, or a “virtual vehicle identification number” (VVIN)? We already have digital object identifiers (DOIs) for electronic documents and other forms of content, a form of persistent identification, with the goal of permanently and unambiguously identifying the object with which a given DOI is associated. But what about an avatar that is currently used by a human operator, namely by functionally and phenomenologically identifying with it? Should we dynamically associate a “digital subject identifier” (DSI) with it? (Madary and Metzinger*, *2016**, p. 17)*.

Maybe there can be a technological solution to this problem, perhaps similar to the RSA cryptosystem. This presents a technical question to mathematicians and computer scientists: What would be a “non-hackable” mechanism for reliably identifying the current user(s) of a given avatar? But even if we find such a mechanism, the epistemological problem of other minds remains. Even if I can be convinced of the identity of an agent I encounter in VR in a way that suffices for all practical and legal purposes, I will still be interested in a higher degree of certainty when it comes to more direct interpersonal relationships in social VR. Interestingly, as regards the personal identity of social others nothing short of absolute certainty seems to be what we are really interested in—although, as one might certainly argue, even in “normal” non-VR scenarios there always remains room for other-person skepticism, because the mere logical possibility of misrepresenting personal identity can never be fully excluded.

There is one variant of the personal-identity problem which could soon become relevant and for which cooperation between VR specialists and philosophical ethicists will be important. Let us conceptually distinguish between an “avatar” as a digital representation of a single human person in VR (over which they can have agentive control and ownership, functionally as well as on the level of conscious experience), a “human agent” as a normal, self-conscious human being currently controlling a biological body outside of VR, and a “virtual agent” as a virtual character or person-model that is computer-controlled, for example by an advanced AI. For human users in VR, it may be impossible to distinguish between avatars and virtual agents, that is, between digital representations of single human persons currently controlled by a real, biological human, and such representations which are actually AI-controlled, for example by an artificial system not possessing self-consciousness and which does not satisfy the current human criteria for personhood (Dennett, 1988). This may at first seem as just an extension of the problem in current online computer games where there is a mixture of non-playable characters and other people, but there will be much more at stake in future contexts generated by the technological confluence of VR and autonomous AI-systems. Again, a technological solution to this problem would be important to prevent social hallucinations, consumer manipulation, or successful deception by malevolent AI systems. But there are looming conceptual complexities.

For example, avatars could also be jointly controlled by distributed groups of human beings (creating problems of legal personhood, accountability, and ethical responsibility). There could be digital person-models that are avatars and virtual agents *at the same time*, because they are simultaneously human-controlled and computer-controlled (HAVAS, see Box 1; for example, resembling a self-consciously controlled biological body possessing a large number of highly intelligent, but unconscious motor subroutines), and perhaps in the future human agents outside of VR could be partly computer-controlled as well. I will not discuss any of these complexities here, but simply point out that the problem of personal identity in VR poses challenges for ethics and legal philosophy and that, on a psychological and cultural level, it may greatly change the landscape of future social interactions.

Although the exact etymological origin of the Latin concept of *persona* is still controversial, it originally referred to the masks worn by actors on stage. It is interesting to note how avatars are exactly this: ever more complex virtual masks worn by human actors on a virtual stage. Social VR resembles an on-stage experience, involving encounters with unknown actors. In this wider context, it may be helpful to recall how in 1938, Antonin Artaud, in first introducing the concept of “virtual reality” described the illusory nature of characters and objects in the theater as “*la réalité virtuelle*” (in a collection of essays entitled *Le Théâter et son double*) while at the same time another classical metaphor for human consciousness is the “theater model of mind” (Baars, 1997a,b; for a critique of this model, see Dennett, 1991; Dennett and Kinsbourne, 1992). Isolating the necessary and sufficient conditions for determining the identity of the person behind any such *virtual persona* is one of the most interesting epistemological problems for philosophers, but they will need help from the VR community in determining what is technologically possible, what is not, and what are rational, evidence-based strategies for risk minimization [the issue will certainly generalize to human interaction with intelligent agents categorized as non-persons, e.g., as a result of future AI/VR confluence, see section Example 7: The “Postbiotic Social Boot-Strapping Scenario” (PSBS)].

## Metaphysics

VR can be interestingly described as a computationally implemented ontology (Heim, 1994, 2000; Chalmers, 2017). Its virtual character consists in the quality of its entities having their attributes without sharing a (real or imagined) physical form, but solely by creating a functional emulation of real objects. On a more abstract level of analysis, virtual realities are functional structures defined by input/output relations and by internal relations between states with different and often complex causal roles. Via interfaces enabling sensorimotor interaction, they have the potential to causally enable the instantiation of specific phenomenal properties in the brains of human users. When implemented and in direct causal interaction with an embodied human user, they can perhaps also be interestingly described as explicit assumptions about *what exists*, as a new type of “metaphysical affordance”: I *can* take this for real. VR opens a space of possible existence assumptions. In providing an explicit model of reality to the user it can also represent objects, properties, and spatial and temporal relations, offer concrete affordances for action, or even present other agentive selves to this user, making them available for reliable and systematic social interaction.

But the novel space of causal interaction opened by VR is not limited to providing affordances for sensorimotor engagement. With the help of advanced brain-computer interfaces (BCIs) we can imagine “mental” actions bypassing the non-neural body (see section Example 5: Walking Around in Your Own NCC With the Help of rt-fMRI-NCCF). Similarly, we can imagine much more causally direct forms of intersubjective communication using much more disembodied forms of social cognition, perhaps even making the “mental” states of users an explicit element of VR (see section Social and Political Philosophy: The danger of complex social hallucinations). As such they could be mutually manipulated, with the interaction acquiring a causal force on its own. Additionally, in VR, assumptions about *what exists* need not obey the physical laws governing our world: In principle, the set of worlds defined by a given level of VR technology will often be much larger than what would be nomologically possible in the actual world. On the other hand, obvious constraints of technological feasibility again strongly compress the space of mere logical possibility.

VR clearly opens new spaces of causal interaction for human agents, but its relevance to philosophical metaphysics is not immediately obvious. Imagine an empty room with a connected headset lying on a table while a computer is running a complex virtual reality demonstration. It would be hard to construct any metaphysical mysteries in this situation. For example, speaking of “virtual objects” or even “virtual worlds” being created by the machine would not justify assuming that just the running of the system itself changes physical reality in any interesting sense. No new building blocks of reality are created.

VR only becomes philosophically interesting when causally coupled to the pre-existing conscious model of reality running in a user's biological brain (Clowes and Chrisley, 2012, p. 511). Then it begins to change the *phenomenal* ontology underlying the user's subjective experience, and of course many unconscious expectations as well. In particular, certain high-level priors and assumptions about what the true causal sources of current sensory input really are may now begin to change as the model containing them is continuously updated [see section Example 1: Consciousness (point 1)]. What would we say if an entirely unconscious, but highly complex and intelligent robot began to interact with a VR system? Would we assign any special metaphysical status to the unconscious internal ontology that emerges as the robot learns to successfully interact with the VR? Obviously, we would not want to say that any relevant new metaphysical entities have been created. What has changed is a model, not the deep structure of the physical world. Call this the “Principle of Metaphysical Irrelevance”: VR technology *per se* does not create any new “virtual objects” in a metaphysically interesting sense. But what about virtual *subjects*? I think the “Principle of Metaphysical Irrelevance” may be interestingly different or invalid in the case of *social ontologies*: What if independent groups of intelligent, virtual agents began to internally model their social relationships in the way human beings do, creating a robust form of virtual intersubjectivity (see Example #7 below)?

The general principle is that to have an ontology is to interpret a world: The human brain, viewed as a representational system aimed at interpreting our world, possesses an ontology too (Metzinger and Gallese, 2003). It creates primitives and makes existence assumptions, decomposing target space in a way that exhibits a certain invariance, which in turn is functionally significant. It continuously updates its model of reality, minimizing prediction error (Friston, 2010; Wiese and Metzinger, 2017). There are explicit and implicit assumptions about the structure of reality, which at the same time shape the causal profile of the brain's motor output and its representational deep structure. But very often in VR, a completely different world needs to be interpreted and predicted by the brain. Thus, an alternative causal structure has to be extracted. For example, the human motor system normally constructs goals, actions, and intending selves as basic constituents of the world it interprets. It does so by assigning a single, unified causal role to them, and empirical evidence demonstrates that the brain models movements and action goals in terms of multimodal representations of organism-object-relations. Obviously, such relations can undergo dramatic changes in VR as the brain continually adapts a hierarchically structured model of reality to the external invariances provided by artificially created input. But the ontology of the human brain, even when causally embedded in an alien media environment, always remains a *representation* of the likely causal structure of the world. It is just the current best guess about this causal structure.

In sum, I think that analytical metaphysics is likely the area in philosophy where we can expect the least fruitful interaction with the VR community, simply because virtual ontologies are orthogonal to philosophical problems in metaphysics. Nevertheless, for philosophers interested in metaphysics, there may still be many interesting issues. These issues include the relationship between possible-world theory and VR; the status of properties, categories, universals, individuals, abstract and fictitious objects, events and selves when epistemically accessed under a VR-mode of presentation; questions about virtual time and virtual space [section Example 1: Consciousness (point 2)]; and perhaps also the promise of a richer and more precise account of what actually constitutes a *Lebenswelt* [Box 1; I return to this issue in section Example 7: The “Postbiotic Social Boot-Strapping Scenario” (PSBS)]. Maybe progress on this traditional concept can be achieved as we now begin to construct entirely new life-worlds from scratch. I will also give one example of unexpected metaphysical contact points between VR and intercultural philosophy in the final section on “Comparative Philosophy.”

## New subfields: digital aesthetics, recent philosophy of technology, and media theory

There are many newer areas of philosophical research for which VR is an obviously central target, including aesthetic judgement and experience (Shelley, 2015), the philosophy of digital art (Thomson-Jones, 2015), the philosophy of technology (Franssen et al., 2015, section 4.1 in Gualeni, 2015), and media philosophy (Gualeni, 2015, Ch. 7; Heim, 2000; Sandbothe, 2000; de Mul, 2015). There already exists a growing literature on VR in these fields, and the interested reader may find entry points to the relevant debates in the works cited here.

## Action theory, free will, and self-consciousness: novel affordances for action

Increasingly, avatars are not just dynamic, user-controlled models of bodies in space. They also begin to enable sensory perception and instantiate complex properties like emotional expression, intelligent gaze-following, and natural language production. Avatars are gradually turning into semi-autonomous, user-controlled models of virtual *selves*. Above, we conceptually distinguished between avatars and virtual agents, but it is certainly conceivable that digital representations of persons emerge that fall under both concepts simultaneously. One philosophically as well as technically interesting aspect lies in the prediction that virtual agents will, by being coupled to artificial intelligence (AI), gain strong *cognitive* self-models. For example, they could function as complex output devices or personoid interfaces by which larger AI systems communicate with humans. But to be really good interfaces, they will have to model the needs and goals of their human users and engage in advanced social cognition. If they reflexively apply their social cognition modules to themselves, they may therefore begin to represent themselves as “knowing selves,” even if they are still partly human-controlled. Therefore, it is also conceivable that such hybrid avatar/virtual agent-systems (Box 1) become proper epistemic agents (EAMs; cf. Metzinger, 2017, 2018a; Box 1) rather than merely virtual bodies moving in virtual space, thereby simulating a system that possesses and actively expands its own knowledge.

A second important aspect of this historical development is that there already exists a biologically grounded self-model in the human operator's nervous system. The human nervous system generates another virtual self-model which often includes an EAM. It has been optimized over millions of years of biological evolution and possesses unconscious as well as conscious content layers. Today, avatars are mostly causally coupled to the phenomenal self-model (PSM; Metzinger, 2003a, 2008) in human brains, but this may change in the future. First pilot studies (Cohen et al., 2012, 2014a,b) demonstrate that, via virtual or robotic re-embodiment, elements of VR can turn into dynamic components of extended self-representation, which not only co-determine locally instantiated phenomenal properties in the human brain, but also enable historically new forms of action. Let us therefore look at novel, philosophically relevant affordances for action potentially provided by VR.

### Example 5: walking around in your own NCC with the help of rt-fMRI-NCCF

In consciousness research (Metzinger, 1995, 2000), a standard background assumption is that in the domain of biological creatures and for every form of conscious content there exists a *minimally sufficient neural correlate* (NCC; see Chalmers, 2000, for a definition, and Fink, 2016, for a refined account). At every point in time, there will also be a minimally sufficient *global* NCC (see Box 1): the set of neurodynamical properties which fully determines the content of subjective experience at this very instant and which has no proper subset of properties that would have the same effect. Let me draw attention to, and at the same time propose, a highly specific application of VR technology here. One technological possibility that will be of great interest for philosophers would be a highly selective combination of VR and neurofeedback generated by real-time functional magnetic resonance imagining, but explicitly targeting the global NCC only.

Let us call this “rt-fMRI-NCCF” (see Box 1). This would be a variant of real-time fMRI-based neurofeedback, but employing VR technology and specifically targeting the neural basis of consciousness. Thus, a real-time fMRI representation of the global NCC would be directly converted into a virtual reality environment: a perceivable dynamic landscape which the conscious subject could passively observe, in which it could navigate, and with which it could then causally interact in entirely new ways. Of course, the VR-based form of rt-fMRI-NCCF I am proposing here would never be “real-time” in any more rigorous conceptual sense, but it would generate historically new forms of self-awareness and afford completely new types of phenomenological self-exploration, including a model-based control of one's own conscious experience (Flohr, 1989; Jacquette, 2014). At present, the only neurophenomenological configuration that comes close to rt-fMRI-NCCF is the stable lucid dream of a scientifically informed person. The stable lucid dream is a conscious state in which the experiential subject *knows* that everything it feels and sees is determined by the NCC currently active in the sleeping physical body, but unlike the potential rt-fMRI-NCCF, it lacks an external, technically realized feedback loop (Metzinger, 2003a, 2013c; Windt and Metzinger, 2007; Voss et al., 2014).

### Example 6: PSM-actions

PSM-actions are all those actions in which a human being exclusively uses the conscious self-model in her brain to initiate an overt action. Of course, there will have to be feedback loops for complex actions, for instance, adjusting a grasping movement in real-time when seeing through the camera eyes of a robot (something still far from possible today). But the relevant causal starting point of the entire action is now not the flesh and bone body, but only the conscious self-model in our brain. In PSM-actions, we simulate an action in the self-model—in the inner *image* of our body—and a machine performs it.

Such experiments are interesting for philosophers, because they touch conceptual issues like action theory, agentive self-consciousness, free will, ethical responsibility, and culpability in a legal sense^3^. On one hand, it is obvious that the phenomenal self-model (PSM) often is a crucial part of a control hierarchy: it is an abstract computational tool for sensorimotor self-control. The PSM is a means to predict and monitor certain critical aspects of the process in which the organism generates flexible, adaptive patterns of behavior and also enables a degree of veto control. On the other hand, it is highly plastic: several representations of objects *external* to the body can transiently be integrated into the self-model. In tool use, a hammer or pliers could be such an object, but rubber hands can demonstrate that the whole process can also take place in a passive condition, by bottom-up multisensory integration alone. For tool-use, “control by embedding” may be a general principle—tools are extensions of bodily organs that need to be controlled to generate intelligent and goal-directed behavior. Whenever the physical body is extended by sticks, stones, rakes, or robot arms, the virtual self-model must be extended as well. Only if an integrated representation of the body-plus-tool exists can the extended system of body-plus-tool in its entirety become part of the brain's predictive control hierarchy. How else *could* one learn to intelligently—i.e., flexibly and in a context-sensitive manner—use a tool, *without* integrating it into the conscious self?

As I have explained elsewhere (Metzinger, 2003a, 2009a), human beings possess physical and virtual organs at the same time. The conscious self-model is a paradigm example of a virtual organ, allowing us to *own* feedback loops, to initiate control processes, and to maintain and flexibly adapt them. What is new is that whole-body surrogates now increasingly provide the human brain with new affordances for action, either as virtual avatars or as physical robots coupled to the virtual self-model in the biological brain. Some element of the expanded control circuit are physical (like the brain and tools), others are virtual (like the self-model and the goal-state simulation). Robots are physical tools; avatars are virtual bodies. It is therefore possible to transiently embed them into the PSM and thereby causally control them “directly out of one's own mind.”

If this general perspective is correct, then we have a maximally parsimonious strategy to scientifically explain self-consciousness without assuming an ontological entity called “the self.” Prediction, testing, and explanation can take place in a much more parsimonious conceptual framework, namely, by introducing the concept of a “transparent self-model” (a conscious model of the person as a whole, which cannot be experienced *as* a model). VR technology is relevant because it offers instruments for experimental testing by selectively influencing different representational layers of the human self-model: bodily self-location, perspective-taking, motion experience, affective self-representation, and so on. One role of VR researchers could be to develop new instruments by which causal dependencies and hypothetical double dissociations between such representational layers can be tested, demonstrated, and technologically exploited, for example by new clinical applications. The maximal realization or “perfect avatar” would be one in which the user can precisely select what aspects of his or her conscious self-model she wants to change by identifying with a digitally created self-representation.

Directly coupling a human PSM with an artificial environment is an example for a new *type* of consciousness technology, one that might even be called a “technology of the self” (section 4.3 in Gualeni, 2015). Currently the effects are still weak, and there are many technical problems. However, it is possible that technological progress will happen faster than expected. What would we do if systems for virtual or robotic re-embodiment became able to function fluidly, with many degrees of freedom, and in real-time? What new conscious states would become possible if one were also able to control *feedback* with the help of a computer-aided brain stimulation directly aimed at the user's self-model, again bypassing the non-neural body? What historically new forms of intersubjectivity and social cooperation could emerge if it were suddenly possible to simultaneously connect several human persons and their self-models via coupled brain computer interfaces, and perhaps even to *merge* them?

## Social and political philosophy: the danger of complex social hallucinations

The number of contact points between VR technology and political philosophy is too large to even begin creating a short list. The convergence of VR and existing social networks may lead to new forms of machine-based manipulation in the formation of political will, novel threats to privacy and autonomy, and a belittlement of the actual political process outside of VR (section 3.2 in Gualeni, 2015). Perhaps most importantly, it is conceivable that what today we call “real life outside of VR” or “the real cultural/historical/political process unfolding in the actual world” would become increasingly experienced as just one possible reality among many others. This might incrementally lead to a dangerous trivialization of real-world suffering and an unnoticed, implicit relativism with respect to value judgements in the original sphere of social interaction (in which any VR technology is still grounded). I would like to term this risk “VR-induced political apathy,” brought about by creeping psychological changes caused in users by a toxic form of mental immersion into a novel medium that originally held the promise to *facilitate* and *enhance* the democratic process. As Stefano Gualeni puts the point:

The interactive experiences of virtual worlds, together with their characteristic combinatorial and procedural processes, can in fact be seen as both

- facilitating and encouraging individual engagement in the socio-political sphere, and
- *denying and confusing the ontological superiority of the world indexed as actual over a myriad of virtual ones. This levelling of value comes with a momentous belittlement of the historical process and of existence itself*.

*Understood from the proposed perspective, all virtual worlds can be deemed as holding an implicit political relevance that is a derivation of their combinatorial, modular, and self-organizing constitution. Both the use and the design of virtual worlds as means of production are, thus, implicitly political activities* (Gualeni, 2015, p. 129).

We may well live through a historical transition that we are only beginning to understand. In the beginning, avatars were just moving statues, models of bodies in time and space. As they have become more realistic, new features like the functionality of gaze-following or emotional expression via facial geometry have been added. It is interesting to note how even at this early stage of VR technology what I have termed “social hallucinations” (see Box 1) are emerging: In users, the phenomenology of “presence” can now be enhanced by a phenomenology of being *socially* situated. Users are confronted not only with virtual models of other bodies, but with actual selves—other agents who are autonomous subjects of experience, mutually sharing an *intersubjective* phenomenology of presence. The classical “place illusion” (section 1.3 in Slater and Sanchez-Vives, 2016) is now complemented and strengthened by an “other-minds illusion.”

My first point is that such social hallucinations will soon become increasingly sophisticated and thus much stronger. One research target is the interaction of the place illusion with the other-minds illusion: how strong is the causal interdependence between spatial immersion and social immersion? If we imagine human users communicating with advanced AI systems in natural language and via anthropomorphic (or at least person-like) interfaces, then we will soon reach a stage where unconscious machines are automatically modeled as independent cognitive agents by the human brain. We may have no control over this process. If so, virtual agents will automatically be experienced as thinkers of thoughts, and the human brain will inevitably begin to predict their behavior as belonging to systems possessing high-level psychological properties like episodic memory, attentional control, and self-consciousness. The combination of VR and AI may therefore lead to a situation in which VR-based anthropomorphic interfaces begin to target the naturally evolved modules for social cognition and agent detection in the biological brains of their human users in intelligent and ever more successful ways. Self-optimizing, but entirely unconscious AI/VR-systems might discover that it simply is most *efficient* to be perceived as self-aware cognitive agents by humans, consequently creating robust and complex social hallucinations as a new phenomenological foundation for man-machine communication.

For empirical researchers in the field of social cognition, this will be of great interest, because it allows for highly innovative and precisely controllable forms of experimental design. The maximal model would be one in which the user's other-mind illusion can be created by every virtual entity she encounters during her VR experience. For philosophers, the impact will extend beyond obviously relevant classical topics like the other-minds problem, social ontology, or political philosophy. The combination of social VR and AI will also touch many issues in applied ethics, including: What is the proper ethical assessment of deliberately causing social hallucinations in human users? Are there ethically recommendable, non-paternalistic applications of VR-based other-minds illusions (see section Applied Ethics)?

### Example 7: the “postbiotic social boot-strapping scenario” (PSBS)

Let me end this section by briefly describing what I think is the most interesting conceptual possibility from a philosophical perspective. I call it the “postbiotic social boot-strapping scenario” (PSBS; see Box 1), and it would again involve a combination of VR and AI.

Let us assume that future AI systems have begun using avatars—VR-based person-like interfaces—to communicate with humans. Non-persons communicate with persons via person-*models*. Through advanced user modeling those systems will have learned how to cause the most reliable and robust social hallucinations in their users, thereby optimizing their overall functionality. Now the crucial assumption behind the PSBS is the logical possibility that such combined AI/VR-systems begin to mutually cause social hallucinations *in each other*. This would occur by the systems applying the algorithms they originally developed for man-machine interaction to machine-machine communication, but still using individual virtual avatars as their interfaces. What I call the “social boot-strapping scenario” would begin when such systems attempt to cause social hallucinations in other AIs as well as in humans. It is conceivable that the continued optimization of combined AI/VR-systems would generate second-order virtual agents (see Box 1), that is, virtual entities that not only possess an internal model of themselves in order to control their behavior, but also harbor a functionally adequate misrepresentation of themselves *as being socially situated*. A first-order virtual agent would be controlled by an AI that uses it as a communication interface. A second-order virtual agent would be driven by a different self-model: it would falsely represent other AIs/virtual agents as real, self-conscious entities. It would represent itself as standing in genuine social relations—as a genuine subject embedded in a network of intersubjective relationships. It is also plausible to assume that such forms of functionally adequate misrepresentation might make groups of virtual agents and groups of interacting AI systems much more efficient—the added explicitly social layer of self-optimization could enable a new level of complexity that would serve to gradually improve the intelligence of the newly emerged overall system. Such second-order agents would therefore not only cause robust social hallucinations in their human users, but also in the AIs controlling them. Groups of such intelligent virtual agents using personoid avatars as their interface or “outward appearance” would instantiate a new property—“virtual intersubjectivity”—by drawing on and mimicking algorithms and neural mechanisms which first appeared in the psychological evolution of biological organisms, were later optimized in man-machine communication, and are now virtually implementing certain types of social cognition and functionally adequate forms of self-deception in postbiotic systems.

Therefore, they would necessarily begin to represent each other as sharing a common *Lebenswelt*. A virtual *Lebenswelt*—or “life-world”—is a pre-given social world in which subjects experience themselves as being united by a primordial quality of “togetherness,” as inhabiting a universe which is no longer “merely virtual,” but rather intersubjectively given. A *Lebenswelt* is co-constituted by a shared first-person plural perspective, by a mentally represented “we,” and is therefore absolutely real and self-evident for every individual virtual agent. To put the point differently, while human beings might still describe the internal social context generated by the interaction of combined AI/VR-systems as “virtual” or “simulated,” these systems *themselves* might evolve a fully transparent representation of their own life-world and accordingly arrive at very different epistemological conclusions about the social context in which they evolve.

This is an example of a new field where philosophers working on theories of social cognition and intersubjectivity could very fruitfully interact with researchers in VR, creating simulated “toy societies.” Here is the most provocative question: Is our own, human *Lebenswelt* ultimately a biologically evolved variant of the PSBS? Is it based on functionally adequate misrepresentations enabling biological organisms to “hallucinate selfhood into each other”? Can we model the relevant transition in virtual agents? *Should* we attempt to do this, or would we be ethically required to relinquish such research pathways altogether? Clearly, such research may be ethically problematic, because it may lead to artificial suffering or a dangerous and irreversible intelligence explosion in autonomously self-optimizing social systems.

## Applied ethics

Less dramatically, VR technology has the potential to increasingly *change* what many philosophers, including Edmund Husserl and Jürgen Habermas, have traditionally called the “life-world” of human beings. As explained above, a life-world is partly constituted by a prescientific, collective phenomenology of intersubjectivity. This underlying phenomenology in turn gives rise to apparently self-evident cultural systems and normative orders which attempt to give a meaning to life and to the shared social institutions that stabilize patterns of collective action. These patterns causally influence psychological properties, determine the content of seemingly individual cognitive processes, and may even shape our personality structure. Elsewhere, I have argued that because of this, VR technology will function as a new cognitive niche to which the human mind will adapt:

*What is historically new, and what creates not only novel psychological risks but also entirely new ethical and legal dimensions, is that* ***one*** *VR gets ever more deeply embedded into* ***another*** *VR: the conscious mind of human beings, which has evolved under very specific conditions and over millions of years, now gets causally coupled and informationally woven into technical systems for representing possible realities. Increasingly, it is not only culturally and socially embedded but also shaped by a technological niche that over time itself quickly acquires a rapid, autonomous dynamics and ever new properties. This creates a complex convolution, a nested form of information flow in which the biological mind and its technological niche influence each other in ways we are just beginning to understand. It is this complex convolution that makes it so important to think about the Ethics of VR in a critical, evidence-based, and rational manner (Madary and Metzinger*, *2016**, p. 20)*.

VR technology poses many new problems for applied ethics, ranging from unexpected psychological risks to military applications. Rather than exploring the ethical and sociocultural ramifications of VR here, I instead refer readers to the first Code of Ethical Conduct Michael Madary and I developed (Madary and Metzinger, 2016). Applied ethics is a prime example of another domain of philosophical research that is of highest relevance for researchers in the field of VR, consumers, and policy-makers alike.

## Philosophy of religion

VR, if applied as a conceptual metaphor in different domains of inquiry, possesses great heuristic fecundity. We have already seen that there are considerable commonalities linking VR and the phenomenon of conscious experience (section Example 1: Consciousness). Religious faith is another example of a domain in which unexpected analogies can be discovered. Religious faith dramatically changes the model of reality under which a human being operates, because it installs or superimposes a new virtual ontology. We can view the evolution of religion as an evolution of pre-technological *augmented reality* (AR) systems aimed at expanding the phenomenology and motivational structure of human beings. Can VR and AR be used as fruitful conceptual metaphors for the philosophy of religion? Let us take a look.

### Example 8: having faith as biosocially evolved augmented reality

In standard situations, the perceptual phenomenology of human beings is largely determined by top-down predictions colliding with the sensory input generated by continuous embodied interaction with an external environment (Friston, 2010). Augmented reality adds an environmental layer that is invisible for others, superimposing a new and additional set of priors onto the conscious subject's model of reality. A novel perspective on organized religion emerges from this: religious-belief-as-enculturated-augmented-reality, where religion is a set of representational functions, originally realized by cultural practices like burial rites, ancestor cults, prayers, sermons and increasingly complex rituals. Three obvious and well-documented adaptive advantages provided by this set of functions are (a) offering a viable psychological strategy for mortality-denial, (b) increasing social cohesion in the context of in-group/out-group conflicts, and (c) the stabilization of existing social hierarchies. As a crude analogy, religious faith is like a metaphysical version of Pokémon Go: it populates the subject's life-world with invisible beings like Gods, angels, and spirits, thereby causally enabling new forms of social hallucination and self-deception (Trivers, 2000). Within a given evolutionary or cultural context, such virtual expansions of a pre-given conscious model of reality may prove to be functionally adequate. Having a religious faith augments an agent's subjective reality, and it often motivates in-group prosocial behavior.

Another example of a related new concept is “transreality gaming” (e.g., Lindley, 2004). Transreality gaming describes a type or mode of gameplay that combines playing a game in a virtual environment with game-related, physical experiences in the real world and vice versa. In this approach, a player evolves and moves seamlessly through various physical and virtual stages, brought together in one unified game space. Let me ask a question that may, initially, sound overly provocative, but which may later prove to possess great heuristic potential: Is religious practice a form of transreality gaming? Are religious rituals like funerals, ancestor cults, or prayer not attempts at an integration of virtual worlds with a biologically grounded life-world? The notion of “transreality gaming” also gives us a new way of looking at what a conscious human being really is: a biological organism that has evolved into a “transreality interaction platform” by enabling causal interactions *across* physical and virtual reality (Martin and Laviola, 2016). From this perspective, religious practices are a particularly interesting special case of this general principle, governing and stabilizing the “player's” day-to-day interactions with their social environment as well as with their own mind.

Above, I have experimentally framed the evolution of religion as the evolution of *augmented reality* systems aimed at expanding the phenomenology of human beings, hypothetically later enabling successful, scalable cooperation in ever larger groups. I have provisionally defined it as a set of representational functions, originally realized by externalized cultural practices like burial rites, ancestor cults, prayers, sermons and increasingly complex rituals, later internalized into the minds of individual agents. This new perspective, leads to a whole range of interesting questions of whether the same set of functions could also be *technologically* implemented. Could there be religious practice in VR? Would it count as valid from a theological perspective? What about the technological implementation of an individual “VR heaven,” where users encounter a medial environment allowing them to interact with their own ideal self, with an impersonal ideal observer, or with virtual angels, saints, and deities? Could there be “VR churches” giving an individual user a comparable phenomenology and the same psychological effects as real social interactions in an embodied religious context? Can there be technologically mediated “virtual rituals” serving basically the same—or historically new—functions? If so, Slater's and Sanchez-Vives' programmatic idea of “Enhancing Our Lives with Immersive Virtual Reality” could even be extended to the sphere of religious practice.

Please note how the “convolution principle” introduced in section Applied Ethics still holds. Again, what is new, and what creates not only novel psychological risks, but also entirely new soteriological dimensions, is that one virtual reality gets ever more deeply embedded into *another* virtual reality: The conscious mind of human beings, which has evolved under very specific conditions over millions of years might become causally coupled and informationally woven into technical systems for representing possible realities—and these could even be of a religious type. Now, the religious mind is not only culturally and socially embedded, but also shaped by a technological niche, a niche that over time quickly acquires rapid, autonomous dynamics and ever new properties. This creates a complex convolution, a nested form of information flow in which the transcendence-seeking mind and its technological niche influence each other in ways we are just beginning to understand. Religious practice in VR could be one of these ways.

## VR-phenomenology in the context of comparative and transcultural philosophy

The VR-experience has a distinct and unique phenomenological profile. What we currently lack is not only a philosophical meta-theory for VR-phenomenology, but suitable conceptual instruments that help us bring out the essence of what really makes conscious experience in VR so interestingly different. At the same time, an important and strongly growing area of philosophy is comparative philosophy, which aims at bringing together and perhaps even integrating philosophical traditions that have developed in relative isolation from one another and that are defined quite broadly along cultural and regional lines (Wong, 2017). I will conclude this contribution by very briefly pointing to a way in which a central concept of Buddhist philosophy—namely, *suññatā*—could be conceptually connected to a philosophical metatheory of virtual reality.

### Example 9: the phenomenology of emptiness and virtuality

Depending on doctrinal context, the Buddhist notion of “emptiness” or “voidness” has many different meanings. Buddhist metaphysics is radically anti-substantialist and anti-essentialist. In a nutshell, this means that entities are conceptually analyzed as being devoid of inherent existence and as lacking any form of “true inner nature”; what in Western traditions has often been simply called “reality” actually is characterized by metaphysical “hollowness” and indeterminacy as to existence vs. non-existence. My first point here is that exactly the same is true of so-called “virtual objects” and other entities like properties, whole situations, or simulated selves as represented in VR. They are not ontologically self-subsistent (i.e., they cannot independently “stand” or independently hold themselves in existence), and they have no self-sustaining, enduring, or essential inner nature beyond the present moment and the ongoing process of being virtually represented *as such*. They depend not only on a complex network of functional relations implemented in a given computational system, but also on this pre-existing network being causally coupled to the physical brain of a user already endowed with consciousness and self-consciousness. Entities in VR are a paradigmatic example of what Buddhist metaphysics would call “dependent origination”: impermanent phenomena arising out of a fluid dynamic of causal interrelatedness.

Interestingly, there is a semantic connection linking the classical Pali term *suññatā* to the concept of “virtuality” (stemming from the late-medieval scholarly neologism *virtualis*, which in turn preserves elements like “potentiality” and “latency of possibilities” characterizing the original Aristotelian notion of “dynamis”). To see this partial, but philosophically relevant overlap, it is particularly helpful to focus not only on the metaphysical, but also the phenomenological reading of *suññatā*, an absolutely central and classical term, which has been a cornerstone of Buddhist philosophy over many centuries (Williams, 2008).

With a minimalist sketch of the metaphysical background already in hand, let us therefore proceed to the phenomenological level of analysis. The VR-experience has a unique phenomenological profile which is another excellent example of a potential future target for interdisciplinary research, and comparative philosophy may actually help us to see the relevant features more clearly. The phenomenological reading of “emptiness” refers to a specific contemplative mode: a way of consciously experiencing the world and the process of knowing this world as inherently selfless (*anatta*). “Seeing out of emptiness” is a specific mode of phenomenally experiencing the world as not seen by a self-as-subject, an ancient meditative practice, in the words of Jiddu Krishnamurti, of “observing without an observer” (Krishnamurti, 2010). The phenomenological reading of *suññatā* also includes a mode of perception in which one neither adds anything to nor takes anything away from what is present, thereby, as it were, “directly seeing” the qualities of suchness, interrelatedness, and impermanence. In this way, the phenomenological reading of “emptiness” refers to a specific mode of conscious experience that can be described as a choiceless form of pure awareness. This mode does not involve an agentive phenomenal self, and things are experienced *neither* as real *nor* as unreal. Therefore, this way of seeing also bears multiple and subtle relations to what, in Western phenomenology, has been described as the “bracketing” of an explicit existence assumption when philosophically investigating a specific content of consciousness, and is associated with technical terms like *epoché*, “eidetic reduction” or “phenomenological reduction” (Beyer, 2016).

Obviously, I am not saying that *suññatā* describes the phenomenology of a standard VR user today. In VR, there is clearly a phenomenal self, and if the place illusion has been successfully created, the experience of actually “being there” can be transparent and subjectively robust (Blanke and Metzinger, 2009). But the second element, the subjective experience of an environment in VR as being “neither real nor unreal” describes the phenomenology of VR very well. My second, phenomenological, point is that what makes VR phenomenology so special is the subjective quality of metaphysical indeterminacy. The claim is that VR-phenomenology is characterized by a *phenomenology of metaphysical indeterminacy*, meaning that objects and environment in VR are subjectively experienced as neither existing nor non-existing. Put differently, Buddhist philosophy may actually offer the conceptual instruments to describe the properties of interest from a more fine-grained phenomenological perspective on what is most interesting about an immersive VR experience. For many elements of subjectively experienced VR, there really is a distinct phenomenal quality of ontological neither-nor-ness: It is not the case that subjectively experienced elements of VR are *either* phenomenally real *or* phenomenally unreal. Phenomenologically, virtuality *is* emptiness if we describe it as an explicit phenomenal experience of metaphysical indeterminacy. I would like to submit that this is a core aspect of what is philosophically interesting about VR phenomenology and what distinguishes it from ordinary waking states. It is therefore noteworthy that Buddhist philosophy may have already given us the conceptual instruments to describe this in a much clearer and heuristically fruitful way.

Of course, things become much more complicated if we include augmented reality setups, and the constantly changing landscape and temporal distribution of phenomenal opacity versus phenomenal transparency into our investigation. The phenomenology of metaphysical indeterminacy is not all-pervading, it is variable and impermanent. But one may speculate that in the future we might, via controlled experimentation, use VR technology *itself* to make progress on philosophically relevant issues like these. For example, one might think of “contemplative” types of VR technology that explicitly aim at enhancing our lives by making the phenomenal quality of metaphysical indeterminacy more robust, then extending it to the place illusion and the sense of self. But as we have seen, many other options are now on the table. Perhaps the most interesting promise of VR technology lies in supporting rational, evidence-based and empirically informed research programs in philosophical phenomenology, with philosophers in turn providing some conceptual foundations and proposing novel research targets for the VR community.

## Summary

As pointed out in the introduction, this article was mainly intended to be a source of inspiration for an interdisciplinary audience. Contact points and potential future directions for interdisciplinary cooperation between different subdisciplines of philosophy and VR research have been explored, through a series of concrete examples and possible research projects. The areas explored were:

- theories of consciousness and VR;
- embodiment and bodily self-consciousness in VR;
- amnestic re-embodiment (in which the user is unaware of having entered VR);
- the problem of personal identity in VR;
- rt-fMRI-NCCF (i.e., “walking around in the neural correlate of consciousness” by a proposed new variant of real-time fMRI-based neurofeedback employing VR technology);
- PSM-actions (i.e., novel forms of actions exclusively initiated in the conscious self-model which causally bypass the non-neural body);
- Complex social hallucinations and the risk of VR-induced political apathy;
- PSBS (the new logical scenario of “postbiotic social boot-strapping”);
- the applied ethics of VR;
- VR and the philosophy of religion;
- VR-phenomenology as a new field of research.

## Author contributions

The author confirms being the sole contributor of this work and approved it for publication.

### Conflict of interest statement

The author declares that the research was conducted in the absence of any commercial or financial relationships that could be construed as a potential conflict of interest.

## Footnotes

^1^In other writings, I have argued for a moratorium for synthetic phenomenology on ethical grounds. For reasons of space, I exclude this issue here, but see Metzinger (2010), Metzinger (2013a), Mannino et al. (2015), and Metzinger (2018b).

^2^This passage draws on an article in the *Oxford Handbook of Spontaneous Thought*, see Metzinger (2018a).

^3^Consider the following thought experiment adapted from Metzinger (2013a). Imagine you are lying in a scanner, controlling a robot at a distance, seeing through its eyes and even feeling motor feedback when its arms and legs move. Experientially, you completely identify with the robot, while at the same time you are moving freely in a situation in which also other human beings are present. Suddenly the new husband of your ex-wife enters the room. He is the person who, a few months ago, destroyed all your plans and your entire personal life. You again feel the mortification, deep hurt, sense of inner emptiness, and existential loneliness following the divorce. Spontaneously an aggressive impulse arises inside you, and almost simultaneously a brief, violent fantasy of killing him emerges. You try to calm yourself down, but before you can suppress the motor imagery that involuntarily went along with the violent fantasy in your conscious mind, the robot has already killed the man with one single, forceful blow. You regain control and are able to back away a few steps. Subjectively it feels as if you never had a chance to control your behavior. But how can one decide if you—from a purely objective perspective—perhaps still possessed the capability of suppressing the aggressive impulse, just in time? In an ethical sense, are you responsible for the consequences of the robot's actions?

## References

[B1] Baars B. J. (1997a). In the Theater of Consciousness. New York, NY: Oxford University Press.

[B2] Baars B. J. (1997b). In the theatre of consciousness. Global workspace theory, a rigorous scientific theory of consciousness. J. Conscious. Stud. 4, 292–309.

[B3] Barrett L. F. Simmons W. K. (2015). Interoceptive predictions in the brain. Nat. Rev. Neurosci. 16, 419–429. 10.1038/nrn3950 26016744 PMC4731102

[B4] Beyer C. (2016). Edmund Husserl, in The Stanford Encyclopedia of Philosophy, ed Zalta E. N. Available online at: https://plato.stanford.edu/archives/win2016/entries/husserl/ (Winter 2016 Edition).

[B5] Blanke O. (2012). Multisensory brain mechanisms of bodily self-consciousness. Nat. Rev. Neurosci. 13, 556–571. 10.1038/nrn3292 22805909

[B6] Blanke O. Metzinger T. (2009). Full-body illusions and minimal phenomenal selfhood. Trends Cogn. Sci. 13, 7–13. 10.1016/j.tics.2008.10.003 19058991

[B7] Blanke O. Slater M. Serino A. (2015). Behavioral, neural, and computational principles of bodily self-consciousness. Neuron 88, 145–166. 10.1016/j.neuron.2015.09.029 26447578

[B8] Boden M. (2006). Mind as Machine: A History of Cognitive Science. Oxford: Oxford University Press.

[B9] Bortolotti L. (2015a). The epistemic innocence of motivated delusions. Conscious. Cogn. 33, 490–499. 10.1016/j.concog.2014.10.005 25459652 PMC7618694

[B10] Bortolotti L. (2015b). Epistemic benefits of elaborated and systematized delusions in schizophrenia. Br. J. Philos. Sci. 67, 879–900. 10.1093/bjps/axv024 27924116 PMC4990704

[B11] Chalmers D. J. (2000). What is a neural correlate of consciousness?” in Neural Correlates of Consciousness: Empirical and Conceptual Questions, ed Metzinger T. (Cambridge, MA: MIT Press), 17–39.

[B12] Chalmers D. J. (2017). The virtual and the real. Disputatio 9, 309–352. 10.1515/disp-2017-0009

[B13] Churchland P. M. (2005). Functionalism at forty. J. Philos. 102, 33–50. 10.5840/jphil2005102136

[B14] Clark A. (2016). Surfing Uncertainty: Prediction, Action, and the Embodied Mind. New York, NY: Oxford University Press 10.1093/acprof:oso/9780190217013.001.0001

[B15] Clowes R. W. Chrisley R. (2012). Virtualist representation. Int. J. Mach. Conscious. 4, 503–522. 10.1142/S179384301240029X

[B16] Cohen O. Druon S. Lengagne S. Mendelsohn A. Malach R. Kheddar A. (2012). fMRI robotic embodiment: a pilot study, in 2012 4th IEEE RAS & EMBS International Conference on Biomedical Robotics and Biomechatronics (*BioRob*) (Rome: IEEE), 314–319. 10.1109/BioRob.2012.6290866

[B17] Cohen O. Druon S. Lengagne S. Mendelsohn A. Malach R. Kheddar A. (2014a). fMRI-based robotic embodiment. Controlling a humanoid robot by thought using real-time fMRI. Presence 23, S.229–241. 10.1162/PRES_a_00191

[B18] Cohen O. Koppel M. Malach R. Friedman D. (2014b). Controlling an avatar by thought using real-time fMRI. J. Neural Eng. 11:35006 10.1088/1741-2560/11/3/035006 24834973

[B19] Craig A. D. (2009). How do you feel–now? The anterior insula and human awareness. Nat. Rev. Neurosci. 10, 59–70. 10.1038/nrn2555 19096369

[B20] de Mul J. (2015). The game of life: narrative and ludic identity formation in computer games, in Representations of Internarrative Identity, ed Way L. (London: Palgrave Macmillan), 159–187.

[B21] De Oliveira E. C. Bertrand P. Lesur M. E. R. Palomo P. Demarzo M. Cebolla A. (2016). Virtual body swap: a new feasible tool to be explored in health and education, in XVIII Symposium on Virtual and Augmented Reality, ed SVR (Gramado: IEEE), 81–89.

[B22] de Ridder D. (2007). Brain and nerve stimulation for mood enhancement. Philosophica 79, 11–24. Available online at: http://www.philosophica.ugent.be/fulltexts/79-2.pdf

[B23] Dennett D. (1988). Conditions of personhood, in What is a Person? ed Goodman M. F. (Clifton: Humana Press), 145–167.

[B24] Dennett D. C. (1991). Consciousness Explained. Boston, MA: Little, Brown and Co.

[B25] Dennett D. C. Kinsbourne M. (1992). Escape from the cartesian theater. Behav. Brain Sci. 15, 234–247. 10.1017/S0140525X00068527

[B26] Ehrsson H. H. (2007). The experimental induction of out-of-body experiences. Science 317:1048 10.1126/science.1142175 17717177

[B27] Fink S. B. (2016). A deeper look at the “neural correlate of consciousness”. Front. Psychol. 7:1044. 10.3389/fpsyg.2016.01044 27507950 PMC4960249

[B28] Flohr H. (1989). Schwierigkeiten der Autocerebroskopie, in Gehirn und Bewußtsein, ed Pöppel E. (Weinheim: VCH), 61–71.

[B29] Franssen M. Lokhorst G.-J. van de Poel I. (2015). Philosophy of technology” in The Stanford Encyclopedia of Philosophy, ed Zalta E. N. (Fall 2015 Edition). Available online at: https://plato.stanford.edu/archives/fall2015/entries/technology/

[B30] Friston K. (2010). The free-energy principle: a unified brain theory? Nat. Rev. Neurosci. 11, 127–138. 10.1038/nrn2787 20068583

[B31] Gualeni S. (2015). Virtual Worlds as Philosophical Tools: How to Philosophize With a Digital Hammer. Basingstoke: Palgrave Macmillan. 10.1057/9781137521781

[B32] Heim M. (1994). The Metaphysics of Virtual Reality. Oxford: Oxford University Press 10.1093/acprof:oso/9780195092585.001.0001

[B33] Heim M. (2000). Virtual Realism. Oxford: Oxford University Press.

[B34] Hohwy J. (2013). The Predictive Mind. Oxford: Oxford University Press 10.1093/acprof:oso/9780199682737.001.0001

[B35] Jacquette D. (2014). What would a cerebroscope do? J. Br. Soc. Phenomenol. 27, 188–199. 10.1080/00071773.1996.11007150

[B36] Krishnamurti J. (2010). Freedom From the Known. New York, NY: Random House.

[B37] Lenggenhager B. Tadi T. Metzinger T. Blanke O. (2007). Video ergo sum: manipulating bodily self-consciousness. Science 317, 1096–1099. 10.1126/science.1143439 17717189

[B38] Limanowski J. (2014). What can body ownership illusions tell us about minimal phenomenal selfhood? Front. Hum. Neurosci. 8:946. 10.3389/fnhum.2014.00946 25505398 PMC4241829

[B39] Limanowski J. Blankenburg F. (2013). Minimal self-models and the free energy principle. Front. Hum. Neurosci. 7:547. 10.3389/fnhum.2013.00547 24062658 PMC3770917

[B40] Lindley C. A. (2004). Trans-reality gaming, in Proceedings of the 2nd Annual International Workshop in Computer Game Design and Technology (Liverpool: John Moores University), 1–10.

[B41] Madary M. Metzinger T. K. (2016). Real virtuality: a code of ethical conduct. recommendations for good scientific practice and the consumers of VR-technology. Front. Robot. AI 3:3 10.3389/frobt.2016.00003

[B42] Mannino A. Althaus D. Erhardt J. Gloor L. Hutter A. Metzinger T. (2015). Artificial intelligence opportunities and risks, in Policy Papers of the Effective Altruism Foundation, 1–16. Available online at: https://ea-foundation.org/files/ai-opportunities-and-risks.pdf

[B43] Martin K. A. Laviola J. J. (2016). The transreality interaction platform: enabling interaction across physical and virtual reality, in 2016 IEEE International Conference on Internet of Things (iThings) and IEEE Green Computing and Communications (GreenCom) and IEEE Cyber, Physical and Social Computing (CPSCom) and IEEE Smart Data (SmartData) (Chengdu: IEEE). 10.1109/iThings-GreenCom-CPSCom-SmartData.2016.54

[B44] Menary R. (ed.). (2010). The Extended Mind. Cambridge, MA: MIT Press 10.7551/mitpress/9780262014038.001.0001(Ed.).

[B45] Metzinger T. (1991). Das phänomenale Ich als komplexes Makro-Simulat. Selbstbewusstsein, in Ein Sonderfall Von Mentaler Simulation, eds Weibel P. F. Rötzer Strategien des Scheins - Kunst, Computer, Medien (München: Boer), 122–145.

[B46] Metzinger T. (1993). Subjekt und Selbstmodell. Paderborn: Schöningh.

[B47] Metzinger T. (ed.). (1995). Conscious Experience. Exeter: Imprint Academic.

[B48] Metzinger T. (ed.). (2000). Neural Correlates of Consciousness: Empirical and Conceptual Questions. Cambridge, MA: MIT Press.

[B49] Metzinger T. (2003a). Being No One: The Self-Model Theory of Subjectivity. Cambridge, MA: MIT Press.

[B50] Metzinger T. (2003b). Phenomenal transparency and cognitive self-reference. Phenomenol. Cogn. Sci. 2, 353–393. 10.1023/B:PHEN.0000007366.42918.eb

[B51] Metzinger T. (2008). Empirical perspectives from the self-model theory of subjectivity: a brief summary with examples. Prog. Brain Res. 168, 215–245. 10.1016/S0079-6123(07)68018-2 18166398

[B52] Metzinger T. (2009a). The Ego Tunnel: The Science of the Mind and the Myth of the Self. New York, NY: Basic Books.

[B53] Metzinger T. (2009b). Why are out-of-body experiences interesting for philosophers? Cortex 45, 256–258. 10.1016/j.cortex.2008.09.004 19046743

[B54] Metzinger T. (2010). The Ego Tunnel: The Science of the Mind and the Myth of the Self. New York, NY: Basic Books.

[B55] Metzinger T. (2013a). Two principles for robot ethics, in Robotik und Gesetzgebung Robotik und Gesetzgebung, ed Hilgendorf E. G. J. P. (Baden-Baden: Nomos), 247–286

[B56] Metzinger T. (2013b). The myth of cognitive agency: subpersonal thinking as a cyclically recurring loss of mental autonomy. Front. Psychol. 4:931. 10.3389/fpsyg.2013.00931 24427144 PMC3868016

[B57] Metzinger T. (2013c). Why are dreams interesting for philosophers? The example of minimal phenomenal selfhood, plus an agenda for future research1. Front. Psychol. 4:746 10.3389/fpsyg.2013.00746 24198793 PMC3813926

[B58] Metzinger T. (2015). M-Autonomy. J. Conscious. Stud. 22, 270–302. Available online at: http://www.ingentaconnect.com/contentone/imp/jcs/2015/00000022/f0020011/art00013

[B59] Metzinger T. (2016). Suffering, in The Return of Consciousness, eds Almqvist K. Haag A. (Stockholm: Axel and Margaret Ax:son Johnson Foundation), 217–240.

[B60] Metzinger T. (2017). The problem of mental action, in Philosophy and Predictive Processing, eds Metzinger T. K. Wiese W. (Frankfurt am Main: MIND Group), 1–26. 10.15502/9783958573208

[B61] Metzinger T. (2018a). Why is mind wandering interesting for philosophers?” in The Oxford Handbook of Spontaneous Thought, eds Fox K. C. R. Christoff K. (New York, NY: Oxford University Press), 97–111.

[B62] Metzinger T. (2018b). Towards a global artificial intelligence charter, in Should We Fear Artificial Intelligence? ed European Parliament Research Service (Brussels: Scientific Foresight Unit), 27–33.

[B63] Metzinger T. Gallese V. (2003). The emergence of a shared action ontology: building blocks for a theory. Conscious. Cogn. 12, 549–571. 10.1016/S1053-8100(03)00072-2 14656499

[B64] Metzinger T. Wiese W. (eds.). (2017). Philosophy and Predictive Processing. Frankfurt am Main: MIND Group.

[B65] Milgram P. Colquhoun H. (1999). A taxonomy of real and virtual world display integration, in Mixed Reality: Merging Real Virtual Worlds, eds Ohta Y. Tamura H. (Berlin; Heidelberg: Springer), 5–30.

[B66] Milgram P. Takemura H. Utsumi A. Kishino F. (1994). Augmented reality: a class of displays on the reality-virtuality continuum, in SPIE Proceedings Vol. 2351: Telemanipulator Telepresence Technologies, ed Das H. 282–292. 10.1117/12.197321

[B67] Millière R. (2017). Looking for the self: phenomenology, neurophysiology and philosophical significance of drug-induced ego dissolution. Front. Hum. Neurosci. 11:245 10.3389/fnhum.2017.00245 28588463 PMC5441112

[B68] Moore G. E. (1903). The refutation of idealism. Mind 12, 433–453. 10.1093/mind/XII.4.433

[B69] Noë A. (2002). Is the visual world a grand illusion? J. Conscious. Stud. 9, 1–12. Available online at: http://www.ingentaconnect.com/contentone/imp/jcs/2002/00000009/f0020005/1283

[B70] Noel J. P. Pfeiffer C. Blanke O. Serino A. (2015). Peripersonal space as the space of the bodily self. Cognition 144, 49–57. 10.1016/j.cognition.2015.07.012 26231086 PMC4837893

[B71] Nour M. M. Evans L. Nutt D. Carhart-Harris R. L. (2016). Ego-dissolution and psychedelics: validation of the Ego-Dissolution Inventory (EDI). Front. Hum. Neurosci. 10:269 10.3389/fnhum.2016.00269 27378878 PMC4906025

[B72] Ohl S. (2017). Tele-Immersion concepts. IEEE Trans. Visual. Comp. Graph. 1:1 10.1109/TVCG.2017.2767590 29990081

[B73] Petkova V. I. Ehrsson H. H. (2008). If I were you: perceptual illusion of body swapping. PLoS ONE 3:e3832. 10.1371/journal.pone.0003832 19050755 PMC2585011

[B74] Putnam H. (1967). Psychological predicates, in Art, Mind, and Religion, eds Capitan W. H. Merrill D. D. (Pittsburgh: University of Pittsburgh Press), 37–48.

[B75] Putnam H. (1975). Mind, Language, and Reality. Cambridge, UK: Cambridge University Press 10.1017/CBO9780511625251

[B76] Putnam H. (1992). Representation and reality. Philos. Phenomenol. Res. 52, 415–418.

[B77] Revonsuo A. (1995). Consciousness, dreams and virtual realities. Philos. Psychol. 8, 35–58. 10.1080/09515089508573144

[B78] Revonsuo A. (2006). Inner Presence: Consciousness as a Biological Phenomenon. Cambridge, MA: MIT Press.

[B79] Sandbothe M. (2000). Media philosophy and media education in the age of the internet. J. Philos. Educ. 34, 53–69. 10.1111/1467-9752.00155

[B80] Serino A. Canzoneri E. Marzolla M. Di Pellegrino G. Magosso E. (2015). Extending peripersonal space representation without tool-use: evidence from a combined behavioral-computational approach. Front. Behav. Neurosci. 9:4. 10.3389/fnbeh.2015.00004 25698947 PMC4313698

[B81] Shelley J. (2015). The concept of the aesthetic, in The Stanford Encyclopedia of Philosophy, ed Zalta E. N. (Winter 2015 Edition). Available online at: https://plato.stanford.edu/archives/win2015/entries/aesthetic-concept/

[B82] Slater M. Sanchez-Vives M. V. (2016). Enhancing our lives with immersive virtual reality. Front. Robot. 3:74 10.3389/frobt.2016.00074

[B83] Thomson-Jones K. (2015). The philosophy of digital art, in The Stanford Encyclopedia of Philosophy, ed Zalta E. N. (Spring 2015 Edition). Available online at: https://plato.stanford.edu/archives/spr2015/entries/digital-art/

[B84] Trivers R. (2000). The elements of a scientific theory of self-deception. Ann. N.Y. Acad. Sci. 907, 114–131. 10.1111/j.1749-6632.2000.tb06619.x 10818624

[B85] Voss U. Holzmann R. Hobson A. Paulus W. Koppehele-Gossel J. Klimke A.. (2014). Induction of self awareness in dreams through frontal low current stimulation of gamma activity. Nat. Neurosci. 17, 810–812. 10.1038/nn.3719 24816141

[B86] Westerhoff J. (2016). What it means to live in a virtual world generated by our brain. Erkenntnis 81, 507–528. 10.1007/s10670-015-9752-z

[B87] Wiese W. (2017). Action is enabled by systematic misrepresentations. Erkenntnis 82, 1233–1252. 10.1007/s10670-016-9867-x

[B88] Wiese W. Metzinger T. K. (2017). Vanilla PP for Philosophers: a primer on predictive processing, in Philosophy and Predictive Processing, eds Metzinger T. K. Wiese W. W (Frankfurt am Main: MIND Group), 1–18. 10.15502/9783958573024

[B89] Williams P. (2008). Mahayana Buddhism: The Doctrinal Foundations. Oxon: Routledge.

[B90] Windt J. M. (2010). The immersive spatiotemporal hallucination model of dreaming. Phenomenol. Cogn. Sci. 9, 295–316. 10.1007/s11097-010-9163-1

[B91] Windt J. M. (2015). Just in time—dreamless sleep experience as pure subjective temporality, in Open MIND, eds Metzinger T. K. Windt J. M. (Frankfurt am Main: MIND Group), 1–34.

[B92] Windt J. M. (2017). From Indian philosophy to cognitive neuroscience: two empirical case studies for Ganeri's self. Philos. Stud. 174, 1721–1733. 10.1007/s11098-016-0826-9

[B93] Windt J. M. Metzinger T. (2007). The philosophy of dreaming and self-consciousness: what happens to the experiential subject during the dream state?, in Praeger Perspectives. The New Science of Dreaming, Vol. 3, Cultural and Theoretical Perspectives, eds Barrett D. McNamara P. (Westport, CT: Praeger Publishers; Greenwood Publishing Group), 193–247.

[B94] Wong D. (2017). Comparative philosophy: Chinese and Western, in The Stanford Encyclopedia of Philosophy, ed Zalta E. N. (Spring 2017 edition). Available online at: https://plato.stanford.edu/archives/spr2017/entries/comparphil-chiwes/

